# Genomic Insights into Syntrophic Lifestyle of ‘*Candidatus* Contubernalis alkaliaceticus’ Based on the Reversed Wood–Ljungdahl Pathway and Mechanism of Direct Electron Transfer

**DOI:** 10.3390/life13102084

**Published:** 2023-10-20

**Authors:** Evgenii N. Frolov, Sergey N. Gavrilov, Stepan V. Toshchakov, Daria G. Zavarzina

**Affiliations:** 1 Winogradsky Institute of Microbiology, Federal Research Center of Biotechnology of the Russian Academy of Sciences, 60 Let Oktjabrja Pr-t, 7, Bld. 2, Moscow 117312, Russia; sngavrilov@gmail.com (S.N.G.); zavarzinatwo@mail.ru (D.G.Z.); 2 National Research Centre “Kurchatov Institute”, Akademika Kurchatova Sq., 1, Moscow 123182, Russia; stepan.toshchakov@gmail.com

**Keywords:** syntrophic acetate oxidation, syntrophic ethanol oxidation, interspecies electron transfer, DIET, alkaliphiles, genome analysis, the Wood–Ljungdahl pathway, energy conservation

## Abstract

The anaerobic oxidation of fatty acids and alcohols occurs near the thermodynamic limit of life. This process is driven by syntrophic bacteria that oxidize fatty acids and/or alcohols, their syntrophic partners that consume the products of this oxidation, and the pathways for interspecies electron exchange via these products or direct interspecies electron transfer (DIET). Due to the interdependence of syntrophic microorganisms on each other’s metabolic activity, their isolation in pure cultures is almost impossible. Thus, little is known about their physiology, and the only available way to fill in the knowledge gap on these organisms is genomic and metabolic analysis of syntrophic cultures. Here we report the results of genome sequencing and analysis of an obligately syntrophic alkaliphilic bacterium *‘Candidatus* Contubernalis alkaliaceticus’. The genomic data suggest that acetate oxidation is carried out by the Wood–Ljungdahl pathway, while a bimodular respiratory system involving an Rnf complex and a Na^+^-dependent ATP synthase is used for energy conservation. The predicted genomic ability of ‘*Ca.* C. alkaliaceticus’ to outperform interspecies electron transfer both indirectly, via H_2_ or formate, and directly, via pili-like appendages of its syntrophic partner or conductive mineral particles, was experimentally demonstrated. This is the first indication of DIET in the class *Dethiobacteria*.

## 1. Introduction

The phenomenon of syntrophy, widely developed in the microbial world, has attracted the great attention of microbiologists for several decades [1,2,3]. The ability of microorganisms to cooperatively overcome the energy barriers by activating mechanisms of indirect interspecies electron transfer (IIET) [4] or of DIET [2] allows them to obtain energy from organic compounds, for which oxidation is thermodynamically unfavorable at normal conditions. The current knowledge base on syntrophic interactions is largely limited, mainly because there are not many cultured syntrophic organisms in the hands of scientists. Among the available cultures, there are even fewer of those classified as obligate syntrophs, i.e., those that do not grow axenically. These bacteria are even kept in culture collections in binary cultures with a hydrogenotrophic partner, usually a methanogenic archaeon or a sulfate reducer. Therefore, they are taxonomically classified as *Candidatus*.

It is well known that in anaerobic deposits of soda lakes, the sulfur cycle dominates [5]. However, intensive microbiological studies of sulfidogenesis in soda lakes have resulted in the isolation of only one pure culture of sulfate-reducing bacterium (SRB) directly oxidizing acetate [6]. Previously, acetate oxidation accompanied by sulfate reduction in soda lakes had only been demonstrated in two syntrophic associations of *Syntrophomonadaceae* representatives and a lithotrophic SRB. ‘*Candidatus* Contubernalis alkaliaceticus’ was the first haloalkaliphilic bacterium able to oxidize acetate, ethanol, propanol, isopropanol, serine, fructose, and isobutyric acid in co-culture with hydrogenotrophic SRB belonging to genus *Desulfonatronum* [7]. The second member of this family, ‘*Candidatus* Syntrophonatronum acetioxidans’, was active at high salt concentrations in association with a hydrogenotrophic partner of genus *Desulfonatronospira* [8,9]. Interestingly, both bacteria are obligate syntrophs and cannot grow in pure cultures; they utilize the canonical mechanism of IIET via H_2_ during their growth in co-cultures with SRB. According to a recent phylogenomic reconstruction based on 120 single-copy conservative marker genes [10], ‘*Ca.* C. alkaliaceticus’ and ‘*Ca.* S. acetioxidans’ represent a separate order in a newly created class ‘*Dethiobacteria*’, a deep lineage within the phylum Firmicutes_D [11]. In 2016, Sorokin and co-authors described three new genera of obligate syntrophs of the order *Syntrophomonadales* able to oxidize straight-chain fatty acids in association with alkaliphilic methanogens of the genus *Methanocalculus* or alkaliphilic SRB. A methanogenic association containing obligate syntrophic bacteria closely related to ‘*Ca.* C. alkaliaceticus’ (99% 16S rRNA gene sequence similarity) was also described [8]. Thus, the study of the anaerobic microbial communities of soda lakes allowed for isolating several obligate syntrophic associations that carry out the oxidation of acetate and other short-chain fatty acids. This activity indicates the essential role of these organisms in the final stages of organic matter decomposition in such environments.

In our previous work [12], we proved the possibility of syntrophic cooperation between ‘*Ca.* C. alkaliaceticus’ and an anaerobic iron-cycling bacterium *Geoalkalibacter ferrihydriticus* [13]. In our experiments, this binary culture oxidized ethanol in the presence of synthesized ferrihydrite, hydrothermal ferrous mineral siderite (FeCO_3_), or without minerals at pH 9.0. Both ferric and ferrous iron-containing phases were converted to magnetite (Fe_3_O_4_), which is stable in alkaline conditions. We suggested that complete ethanol oxidation and significant enhancement of ‘*Ca.* C. alkaliaceticus’ growth in the presence of siderite occur due to the formation of magnetite, allowing for the consumption of electrons released from ethanol oxidation by ‘*Ca.* C. alkaliaceticus’ via DIET mechanism [12].

‘*Ca.* C. alkaliaceticus’ was the first alkaliphilic syntrophic bacterium shown to participate in syntrophic associations with different hydrogen-utilizing partners, but the mechanisms of interspecies interactions within these associations remained unclear. The goal of the current work was to clarify these mechanisms. Accordingly, we screened the genome of ‘*Ca.* C. alkaliaceticus’ for the determinants of its carbon and energy metabolism. The obtained data helped us to clarify the mechanism of interspecies interactions between ‘*Ca.* C. alkaliaceticus’ and *G. ferrihydriticus* during their growth in the presence of Fe-containing minerals.

## 2. Materials and Methods

### 2.1. Genome Sequencing and Assembly

Genome sequencing and assembly were performed by a hybrid approach using both long-read (Oxford Nanopore Technologies, Oxford, UK) and short-read (Illumina, San Diego, CA, USA) technologies. High molecular weight genomic DNA was isolated from approximately 5 × 10^9^ cells of binary culture using a Genomic tip 20/G column (Qiagen, Hilden, Germany) according to the manufacturer’s instructions for Gram-positive bacteria. The library for Illumina sequencing was prepared with Nextera DNA Flex Library Prep kit according to the manufacturer’s instructions. The Illumina library was sequenced on the MiSeq sequencer using 2 × 250 paired-end sequencing chemistry. A total of 1,266,892 read pairs were obtained. Bad tiles were removed by filterbytile.sh script from the BBMap package (https://sourceforge.net/projects/bbmap/ (accessed on 4 March 2022)). Quality trimming was performed using CLC Genomic Workbench 10.0 (Qiagen, Germany). Finally, 1,087,804 read pairs were used for the assembly. 

The library for nanopore sequencing was prepared with a Rapid Barcoding Kit (SQK-RBK004, Oxford Nanopore Technologies, Oxford, UK). Sequencing was performed using a FLO-MIN-106D SpotON Flow Cell (R9.4) on a GidION X5 sequencer. Basecalling was performed on the instrument. As a result, 119,367 reads were obtained, corresponding to approximately 170× coverage (about 600 Mbases in total). Hybrid de novo assembly was performed with a Unicycler package [14] and resulted in one circular contig of 3,558,949 bp length. 

Genome completeness and complexity were assessed with a lineage-specific marker set (based on 213 protein markers) generated by the ‘lineage workflow’ of the CheckM package [15]. To assess the presence of *Geoalkalibacter*-related sequences in the sample, all reads were mapped to the finalized genome, and unmapped reads were collected. Unmapped reads were either classified using Kaiju metagenomic read classificatory [16] against the NCBI nr database or mapped to the *G. ferrihydriticus* reference genome (NZ_JWJD00000000.1) with CLC Genomic Workbench using default mapping parameters. These two approaches confirmed each other and showed that *G. ferrihydriticus* reads comprised approximately 0.13% of the dataset.

### 2.2. Genome Annotation

Genome annotation was performed by the NCBI prokaryotic genome annotation pipeline (PGAP) [17]. Analysis of CRISPR repeats and Cas protein clusters was performed with the CRISPRCasFinder tool [18]. Search for horizontally transferred genome loci was performed with IslandViewer4 server [19]. Analysis of bacteriophage-related genomic regions was performed using the PHASTER web server [20]. Visualization of the structural alignment of the Cmr5 protein was performed with the ESPript3 web server (http://espript.ibcp.fr (accessed on 28 February 2022)) [21]. To identify the determinants of EET, the genome of ‘*Ca.* C. alkaliaceticus’ was primarily screened for iron metabolism genes using two different bioinformatics approaches, using a bioinformatic tool FeGenie [22] and manually against a set of ca. 70 previously described genes of multiheme cytochromes involved in dissimilatory Fe(III) reduction and energy-producing Fe(II) oxidation, as well as related porins and e-pili assemblies (complete list of the genes is described in Zavarzina et al. [23] and references therein).

### 2.3. Growth Experiments

In this work, the type species of ‘*Ca.* C. alkaliaceticus’ Z-7904^T^ (=VKM B-2362^T^) and *G. ferrihydriticus* Z-0531^T^ (DSMZ = 17461T = VKM B-2349^T^) were used [7,13]. The cell morphology of syntrophic partners differed from each other. Cells of ‘*Ca.* C. alkaliaceticus’ are straight or slightly curved nonmotile rods of 0.5–0.7 × 3.0–7.0 μm, forming terminal endospores. Cells of *G. ferrihydriticus* are short, motile, non-spore-forming rods of 0.3–0.5 × 1.0–2.0 μm. Corresponding information has been added to the text.

To clarify the spatial structure of the interspecies interaction in the co-culture of ‘*Ca.* C. alkaliaceticus’ and *G. ferrihydriticus*, a biofilm experiment was performed using synthesized magnetite and anaerobically prepared basal medium (BM) with the following composition (g l^−1^): KH_2_PO_4_—0.2; MgCl_2_—0.1; NH_4_Cl—0.5; KCl—0.2; NaCl—1.0; Na_2_CO_3_—3.0; NaHCO_3_—10.0; yeast extract—0.05; trace element solution [24]—1 mL l^−1^; ethanol—25 mM. BM was boiled, cooled under a flow of N_2_ (100%), then dispensed into Hungate tubes and autoclaved. No reducing agents were added to the medium to avoid a reaction with magnetite. Synthesized magnetite was prepared by titrating an equimolar mixture of Fe_2_(SO_4_*)*_3_ × 9H_2_O and FeSO_4_ × 7H_2_O salts with 10% NaOH to pH 9.0. Precipitate was washed three times with distilled water.

A thin layer of synthesized magnetite was applied to the cover glass, dried, placed in the 15 mL penicillin flasks under N_2_ flow, then closed with a rubber stopper and aluminum lid and sterilized. Separately sterilized BM (1 mL) was added to the sterile flask with magnetite glasses and then inoculated with ‘*Ca.* C. alkaliaceticus’ and *G. ferrihydriticus* (5% vol). After three weeks of incubation at 35 °C, the liquid medium was removed by opening the flasks, the cover glass was dried, and then the magnetite surface was examined by scanning electron microscopy (SEM). Five flasks were used for the experiment, but only three showed growth.

### 2.4. Microscopy

To investigate the microstructure of the biofilm samples, pieces of cover glasses with a magnetite layer were carefully fixed with double-sided carbon tape, triple-coated with Au on the stages, and then routinely examined by scanning electron microscopy (SEM) on LEO 1450VP (Carl Zeiss, Oberkochen, Germany).

## 3. Results and Discussion

### 3.1. General Genome Properties, Genome Mobility, and Genetic Immunity Systems

Shotgun sequencing and de novo assembly of the binary culture genomes resulted in one circular chromosome of 3,558,949 bp length corresponding to ‘*Ca.* C. alkaliaceticus’. The GC-content of the ‘*Ca.* C. alkaliaceticus’ genome was 41.2%. *G. ferrihydriticus*-related reads represented only 0.13–0.14% of the dataset. This might be explained either by the low abundance of *G. ferrihydriticus* in the binary culture or by the low efficiency of the mild lysis used to extract high molecular weight DNA from *G. ferrihydriticus* cells. Analysis of the ‘*Ca.* C. alkaliaceticus’ genome completeness and contamination with CheckM [15] showed that 196 of 213 protein markers were present in single copies, while nine protein markers were duplicated, resulting in 95.13% and 2.82% of completeness and contamination, respectively. The NCBI prokaryotic genome annotation pipeline [17] detected 3516 genes, including 68 RNA genes, 3383 protein-coding genes, and 65 pseudogenes (Table S1).

Analysis of genomic islands, mobile genetic elements, and phage-related genomic regions revealed that the genome of the ‘*Ca.* C. alkaliaceticus’ can be characterized by a very high level of genome mobility: over 410 kilobases, corresponding to 11.6% of the genome, were detected as genomic islands, including 108.9 kb of bacteriophage-related regions and 89 IS-element-related ORFs (Table S1 and Supplementary File S1). To establish a reason for high susceptibility to invader DNA, determinants of genetic immunity were analyzed using the CRISPRCasFinder web server [18]. A 41 kb genomic region was detected containing three CRISPR arrays with identical repeat sequences and a set of Cas genes, mostly corresponding to the type III system (Table S2). The scaffold sequences of all three CRISPR arrays were identical, suggesting that the whole cluster corresponds to a single CRISPR genetic immunity system. However, an IS3-type mobile element insertion was detected between the Cmr5 (HUE98_14335) and Cmr4 (HUE98_14350) genes. The Cmr5 subunit was seriously affected by this insertion, resulting in the loss of the N-terminal α1-helix (Figure S1), which is critical for the formation of helical core filaments of the Cmr RNA-silencing complex [25]. In addition to this insertion, the analysis of the order and composition of Cas genes in the detected locus gives the impression that the primary structure of the III-B type system was further disturbed by another recombination event, which introduced a fragment of the type I CRISPR-Cas cluster into the locus (Table S2). Considering the recently reported results on enhanced spacer-mediated recombination between phage and CRISPR-cas loci [26], which may lead to the introduction of whole Cas-genes cluster into existing CRISPR-Cas cassettes, there is a possibility that the same scenario occurred in vivo in ‘*Ca.* C. alkaliaceticus’. It should be noted that it is not possible to reliably assess what the primary event was for the process of disruption of the genetic immune system of ‘*Ca.* C. alkaliaceticus’: insertion of the IS3 element into the type III-B cluster or other recombination events. Nevertheless, it can be stated that multiple disruption events of the CRIRSPR-Cas system led to a surprisingly high level of genome mobility of ‘*Ca.* C. alkaliaceticus’.

### 3.2. Genomic Determinants of ‘Ca. C. alkaliaceticus’ Metabolism

‘*Ca.* C. alkaliaceticus’ has been described as a syntrophic acetate-oxidizing bacterium (SAOB) capable of oxidizing acetate, ethanol, propanol, isopropanol, isobutyrate, serine, and fructose in a co-culture with *Desulfonatronum cooperativum* [7]. Anaerobic oxidation of fatty acids and alcohols in syntrophic co-cultures is thermodynamically feasible if only the released electrons are effectively scavenged by syntrophic partners, which in most cases are hydrogenotrophic methanogens or sulfate reducers. Two types of interspecies electron transfer have been described so far [2,27]. In the case of IIET, soluble electron carriers (hydrogen, formate, cystine/cysteine, quinone/hydroquinone, and others) shuttle electrons between electron-donating and electron-accepting partners. DIET involves electron transfer through electrically conductive pili or electrically conductive materials such as conductive Fe minerals, as well as electron transfer between redox-active proteins associated with the outer cell surface [2].

#### 3.2.1. Syntrophic Acetate Oxidation

**Figure 1:**
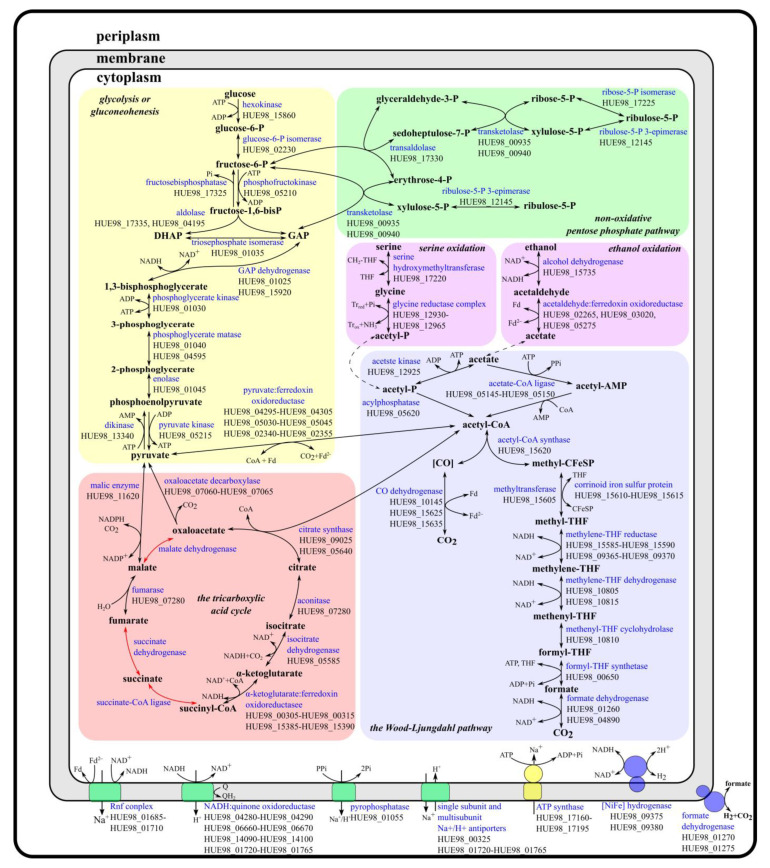
An overview of the metabolism of ‘*Ca.* C. alkaliaceticus’, reconstructed within this study from its genome. Red lines indicate the absence of particular steps of the identified metabolic pathways in ‘*Ca.* C. alkaliaceticus’. Abbreviations: CoA, coenzyme A; DHAP, dihydroxyacetone phosphate; GAP, glyceraldehyde-3-phosphate; Pi, phosphate; PPi, pyrophosphate; THF, tetrahydrofolate; Tr, thioredoxin.

The previously characterized SAOBs (e.g., *Syntrophaceticus schinkii*, *Thermacetogenium phaeum*, or *Tepidanaerobacter acetatoxydans*) use acetate kinase and phosphotransacetylase for the conversion of acetate to acetyl-CoA [28]. The genome of ‘*Ca.* C. alkaliaceticus’ encodes an acetate kinase (HUE98_12925) but not a phosphotransacetylase. Thus, the activation of acetate by acetate kinase and phosphotransacetylase is strongly impaired. However, ‘*Ca.* C. alkaliaceticus’ has the gene for acylphosphatase (HUE98_05620), which could also produce acetyl-CoA from acetylphosphate and thus replace phosphotransacetylase (Figure 1). Moreover, the genome of ‘*Ca.* C. alkaliaceticus’ harbors genes encoding AMP-forming acetate-CoA ligase (HUE98_05145 and HUE98_05150), which catalyze the conversion of acetate to acetyl-CoA via acetyl-AMP (Figure 1). This mode of acetate activation has been proposed for SAOB lacking acetate kinase and/or phosphotransacetylase such as *Pseudothermotoga lettingae*, *Clostridium ultunense*, and ‘*Candidatus* Syntrophonatronum acetioxidans’ [9,28].

The next step is the oxidation of acetyl-CoA. Most of the SAOB taxa described so far belong to the physiological group of acetogens, which possess the Wood–Ljungdahl pathway. This pathway has been experimentally demonstrated to be used in the reverse direction for syntrophic acetate oxidation (SAO) by *Syntrophaceticus schinkii*, *Thermacetogenium phaeum*, and *Tepidanaerobacter acetatoxydans* [28,29]. The genome of ‘*Ca.* C. alkaliaceticus’ contains all genes encoding the Wood–Ljungdahl pathway for the oxidation of acetyl-CoA to H_2_ and CO_2_ (Figure 1). Most of these genes were found in a single cluster, including the genes of methylene-THF reductase (HUE98_15585-HUE98_15590), methyltransferase (HUE98_15605), corrinoid iron–sulfur protein (HUE98_15610-HUE98_15615), acetyl-CoA synthase (HUE98_15620), and carbon monoxide dehydrogenases (HUE98_15625, HUE98_15635). Interestingly, this gene cluster harbors genes for heterodisulfide reductase subunit A (HUE98_15600) and methylviologen-reducing hydrogenase subunit delta (HUE98_15595), which, together with heterodisulfide reductase subunits B (HUE98_11610) and C (HUE98_11615), form the MvhD/HdrABC complex. In the acetogen *Moorella thermoacetica*, MetF and HdrABC/MvhD form a transcript. Partial purification of this enzyme complex from *M. thermoacetica* showed that it is a heterohexamer of MetFV, HdrABC, and MvhD [30]. This complex may be involved in NADH production from methyl-THF oxidation [9].

The genes for methylene-THF reductase (HUE98_09365-HUE98_09370) and carbon monoxide (HUE98_10145-HUE98_10165) dehydrogenases in ‘*Ca.* C. alkaliaceticus’ are multiplicated, and their copies were found in different loci. Other genes belonging to the methyl branch of the Wood–Ljungdahl pathway in ‘*Ca.* C. alkaliaceticus’ were scattered across the genome, away from the main gene cluster described above. These genes include formyl-THF synthetase (HUE98_00650), methenyl-THF cyclohydrolase (HUE98_10810), and methylene-THF dehydrogenase (HUE98_10805, HUE98_10815). 

The genome of ‘*Ca.* C. alkaliaceticus’ also contains three genes encoding formate dehydrogenases. Two of them (HUE98_01260 and HUE98_04890) seem to have the active site intracellularly and formate, and H_2_ can, therefore, be interconverted in the cytoplasm. Next to formate dehydrogenase HUE98_04890 are genes encoding the subunits of NAD(P)H dehydrogenase (HUE98_04875-HUE98_04885) and formate dehydrogenase accessory sulfurtransferase (HUE98_04895). We assume that NAD(P)^+^ is reduced during the formate oxidation. The other formate dehydrogenase consists of two subunits. The alpha subunit (HUE98_01270) has a twin-arginine translocation (TAT) signal, indicating that it is exported outside the cell and, thus, might be involved in extracellular formate conversion. Beta subunit (HUE98_01275) has one C-terminal transmembrane helix, suggesting that it is membrane-anchored. The genome does not encode any formate transporters, and it therefore seems most plausible that ‘*Ca.* C. alkaliaceticus’ produces H_2_ as an end product during SAO. However, it could potentially convert H_2_ to formate outside the cell using extracellular formate dehydrogenases.

#### 3.2.2. Hydrogen Production during Syntrophic Acetate Oxidation

The genome of ‘*Ca.* C. alkaliaceticus’ encodes one [NiFe] hydrogenase consisting of a large (HUE98_09375) and a small (HUE98_09380) cytochrome *c*3-containing subunit lacking signal peptides for translocation across the cell membrane. The small subunit is anchored to the membrane, probably intracellularly. The [NiFe] hydrogenase genes are co-localized with a methylene-THF reductase gene (MetF) and are probably directly involved in acetate metabolism. We propose that [NiFe] hydrogenase oxidizes NADH to form hydrogen, which may serve as an interspecies electron carrier used by syntrophic partners of ‘*Ca.* C. alkaliaceticus’.

#### 3.2.3. Energy Conservation during Syntrophic Acetate Oxidation

The reversal of the Wood–Ljungdahl pathway produces only one ATP molecule during formyl-THF synthetase activity, but acetate activation costs two ATPs. Therefore, energy must come from the generation of a sodium or proton motive force during SAO. The genome of ‘*Ca.* C. alkaliaceticus’ encodes for the Rnf complex (ferredoxin: NAD^+^ oxidoreductase; HUE98_01685-HUE98_01710), which mediates the electron transfer from ferredoxin to NAD^+^, as well as Na^+^/H^+^-translocating pyrophosphatase (HUE98_01055), which hydrolyzes pyrophosphate during acetate activation. We propose that both the Rnf complex and the pyrophosphatase generate an ionic driving force that is used for energy conservation by the F_1_F_0_-ATP synthase (HUE98_17160-HUE98_17195). The membrane-integral c-subunit of F_1_F_0_-ATP synthases determines the ion specificity of the enzyme. The c-subunit of the F_1_F_0_-ATP synthase of ‘*Ca.* C. alkaliaceticus’ has conserved amino acids involved in Na^+^-binding. This finding suggests that the F_1_F_0_-ATP synthase in this organism is Na^+^-dependent. This is an advantage at haloalkaline conditions where extracellular Na^+^ concentrations are high and, thus, contribute to the sodium motive force. The genome of ‘*Ca.* C. alkaliaceticus’ also contains genes encoding for single- and multisubunit Na^+^/H^+^ antiporters (HUE98_00325 and HUE98_01720-HUE98_01765) that keep Na^+^ levels inside the cell lower than outside. Finally, we found the genes encoding putative membrane (HUE98_01720-HUE98_01765) and soluble (HUE98_04280-HUE98_04290, HUE98_06660-HUE98_06670, HUE98_14090-HUE98_14100) parts of NADH:quinone oxidoreductase. This enzyme complex transfers electrons from NADH to quinones, generating a proton motive force.

#### 3.2.4. Syntrophic Ethanol Oxidation

In the first step, ethanol is oxidized to acetaldehyde by alcohol dehydrogenase (HUE98_15735) using NAD^+^ as the oxidizing agent (Figure 1). In the second step, acetaldehyde is oxidized to acetate. There are two possibilities for anaerobic acetaldehyde oxidation [31]. Firstly, via the acetylating acetaldehyde dehydrogenase, phosphotransacetylase, and acetate kinase, one ATP is gained, and one NAD^+^ is reduced. However, the genome of ‘*Ca.* C. alkaliaceticus’ lacks the genes encoding the acetylating acetaldehyde dehydrogenase and phosphotransacetylase. Therefore, we propose that acetaldehyde is oxidized to acetate by acetaldehyde:ferredoxin oxidoreductase (the second way for anaerobic acetaldehyde oxidation), and energy must be conserved by alternative mechanisms, e.g., by oxidative phosphorylation. Three aldehyde:ferredoxin oxidoreductases (HUE98_02265, HUE98_03020, and HUE98_05275) are encoded in the genome of ‘*Ca.* C. alkaliaceticus’. Thus, ferredoxin and NAD^+^ are reduced in a 1/1 stoichiometry during the ethanol oxidation to acetate. We predict that ferredoxin is oxidized by the Rnf complex, which couples the electron transfer from reduced ferredoxin (Fd^2−^) to NAD^+^ with the translocation of Na^+^ across the cytoplasmic membrane (see above). Afterward, NADH is then oxidized to hydrogen by the [NiFe] hydrogenase.

#### 3.2.5. Carbon Metabolism

The genome of ‘*Ca.* C. alkaliaceticus’ encodes all the enzymes of the Embden–Meyerhof glycolytic pathway (Figure 1). Therefore, this organism has the ability to degrade sugars and/or to perform gluconeogenesis, as indicated by the presence of enzymes specifically performing the reverse reactions: pyruvate phosphate dikinase (HUE98_13340) and fructose-1,6-bisphosphatase (HUE98_17325). Examination of the genome revealed that ‘*Ca.* C. alkaliaceticus’ also possesses the non-oxidative branch of the pentose phosphate pathway (Figure 1), which is represented by reversible rearrangement reactions of C4, C5, C6, and C7 phosphosugars. In addition, the genome encodes for an incomplete tricarboxylic acid (TCA) cycle (Figure 1). Genes encoding succinate-CoA ligase, succinate dehydrogenase, and malate dehydrogenase are absent. Therefore, we suggest that the Embden–Meyerhof glycolytic pathway, the non-oxidative branch of the pentose phosphate pathway, and the TCA cycle are part of anabolism, not energy metabolism.

Serine is one of the growth substrates of ‘*Ca.* C. alkaliaceticus’. Serine can be oxidized to glycine by serine hydroxymethyl transferase (HUE98_17220), after which the glycine reductase complex (HUE98_12930-HUE98_12965) converts glycine to acetylphosphate (Figure 1). We assume that acetylphosphate is then converted to acetyl-CoA and metabolized via the reverse Wood–Ljungdahl pathway.

The description of ‘*Ca.* C. alkaliaceticus’ states that the organism grows on propanol, isopropanol, and isobutyrate [7], but its genome lacks the genes encoding the pathways for their utilization. Therefore, these compounds cannot be the carbon sources for ‘*Ca.* C. alkaliaceticus’. However, propanol and isopropanol can be oxidized by alcohol dehydrogenase (HUE98_15735) and aldehyde:ferredoxin oxidoreductases (HUE98_02265, HUE98_03020, and HUE98_05275) due to the promiscuity of these enzymes and thus, these compounds could be the energy substrates in the presence of any other carbon sources. This hypothesis requires further microbiological, proteomic, and biochemical confirmation.

#### 3.2.6. Direct Interspecies Electron Transfer

As mentioned above, DIET could be an essential mechanism for SAOB to dispose of the electrons released from acetate oxidation. This mechanism is provided by the ability of syntrophic partners for extracellular electron transfer (EET), i.e., by the ability of their enzyme complexes and cellular structures to transfer electrons outside the cell to insoluble acceptors, such as Fe(III) minerals and accept electrons from insoluble extracellular donors [32]. Our screening of the determinants of EET in the genome of ‘*Ca.* C. alkaliaceticus’ revealed two different pathways for electron exchange with insoluble Fe(III) oxides in the organism: the ‘canonical’ cytochrome-driven pathway described in various microorganisms respiring Fe(III) [2,33] and a flavin-based EET mechanism recently revealed in pathogenic and syntrophic Gram-positive bacteria [34].

The first EET pathway is determined in ‘*Ca.* C. alkaliaceticus’ by several multihemes related to Fe(III) reduction, including those involved in the extracellular reduction in insoluble Fe(III) forms. These are represented by four different multiheme cytochromes. The major one is a putative outer cell surface octaheme cytochrome encoded by the gene HUE98_14995 flanked by two transcriptional regulators. It shares equally weak homology with putative terminal Fe(III) reductases MtrA of *Shewanella oneidensis* and OmhA of *Carboxydothermus ferrireducens*, as well as with a cell surface cytochrome OmcX of *Geobacter sulfurreducens*. Two other multihemes are homologous to previously described quinol-oxidizing cytochromes. The pentaheme HUE98_13195 shares homology with the CymA protein of *S. oneidensis* and four different putative quinol-oxidizing cytochrome subunits of the CSIM-family enzyme complexes from *C. ferrireducens*. The tetraheme HUE98_12565 is weakly homologous to a putative Q-oxidase subunit CarfeDRAFT_00001220 of a CISM family enzyme complex from *C. ferrireducens*. An additional diheme cytochrome HUE98_12090 of ‘*Ca.* C. alkaliaceticus’ shares no homology with previously described determinants of EET but is homologous to multihemes of unknown function from various halophilic and alkaliphilic bacteria. Also, three homologs of non-cytochrome components of EET pathways described in model Fe(III)-reducers *S. oneidensis* and *G. sulfurreducens* were identified in ‘*Ca.* C. alkaliaceticus’ genome. These are represented by HUE98_04205, which is homologous to the iron–sulfur cluster-binding subunit CbcV of the putative menaquinol:ferricytochrome *c* oxidoreductase complex Cbc3, and by HUE98_15710 homologous to a periplasmic fumarate reductase SO0970. ‘*Ca.* C. alkaliaceticus’ appeared to possess the complete gene set for type IV pili production encoded by the HUE98_10910-HUE98_10975 locus, although the putative major pilin PilA HUE98_10915 is homologous to a ‘long-type’ non-conductive pilin protein of *C. ferrireducens* [35,36].

The second (flavin-based) EET mechanism is determined in ‘*Ca.* C. alkaliaceticus’ by the homologs of a novel type of NADH dehydrogenase Ndh2, encoded by HUE98_05660, the protein DmkB involved in demethylmenaquinone (DMK) biosynthesis; a membrane-bound electron-transferring DMK-oxidizing protein complex EatAB (HUE98_01775- HUE98_01780); its partner electron-accepting lipoproteins FmnB (HUE98_01770) and PplA (HUE98_05615); and the protein complex FmnA-EcfA for FAD secretion (HUE98_02035-HUE98_02045). According to the model of this EET mechanism reported for *Lysteria monocytogenes*, FmnA secretes FAD, which is used by FmnB to post-translationally modify (FMNylate) PplA. EET is achieved by a series of electron transfers from intracellular NADH to the membrane pool of DMK, initiated by Ndh2. Electrons are further transferred from DMK to FMN groups on PplA or free flavin shuttles, possibly via the membrane proteins EetA and EetB, and finally from reduced flavins or FMNylated-secreted PplA lipoproteins to terminal extracellular acceptors [34]. In contrast to *L. monocytogenes*, the genes of this EET pathway in ‘*Ca.* C. alkaliaceticus’ are distributed sporadically across the genome; the organism lacks any homologs of the DmkA membrane protein involved in DMK biosynthesis and possesses ECF transporter ATPase subunits genes encoded in the same locus with FmnA and previously detected in *Lactobacillus plantarum* [34].

It is still unclear whether the flavin-based EET pathway has a function in respiration or in redox control of the environment, for instance, in iron uptake [37]. In ‘*Ca.* C. alkaliaceticus’, the latter function of this pathway could be proposed considering the presence of ‘respiratory’ EET proteins in the organism and identification of the genes determining the acquisition of iron ions and siderophores by the organism (171 homologs of 19 genes known to be involved in iron and siderophore acquisition were identified in ‘*Ca.* C. alkaliaceticus’ using the FeGenie tool).

### 3.3. Biofilm Formation by ‘Ca. C. alkaliaceticus’ and Geoalkalibacter ferrihydriticus in a Binary Culture

**Figure 2:**
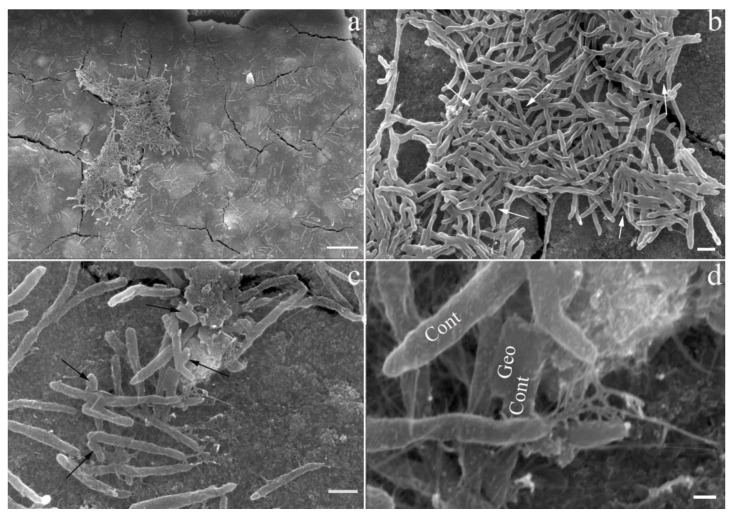
Colonization of magnetite surface by a binary culture of ‘*Ca.* C. alkaliaceticus’ and *G. ferrihydriticus*: (**a**,**b**) formation of colonies by ‘*Ca.* C. alkaliaceticus’ (long curved cells); (**b**) connection of cells and magnetite surface by pili (white arrows); (**c**) cells of *G. ferrihydriticus* (short straight cells, marked with black arrows) closely associated with putative cells of ‘*Ca.* C. alkaliaceticus’; (**d**) pili formed in the binary culture; Geo—a putative cell of *G. ferrihydriticus*; Cont—putative cells of ‘*Ca.* C. alkaliaceticus’. Bar, 10 µm (**a**); 1 µm (**b**,**c**); 200 nm (**d**).

The study of biofilm formation on magnetite surfaces by scanning electron microscopy allowed us to clarify the nature of the relationships between the two syntrophic bacteria (Figure 2a). We confirmed the uneven distribution of bacterial cells on the mineral surface and the tendency to form dense colonies, which was observed previously in the experiments with synthesized ferrihydrite, siderite, or without minerals [12]. Cells with the morphology characteristic for ‘*Ca.* C. alkaliaceticus’ constituted the absolute majority of the biofilm, while putative *G. ferrihydriticus* cells were sporadically represented in the biofilm (Figure 2b,c). We previously observed the same cell-to-cell ratio in syntrophic associations grown with siderite or without Fe-containing minerals [12]. Interestingly, the cells of both bacteria were connected to each other and to the conductive mineral magnetite by pili-like structures, which were about 10 nm thick (Figure 2d). The presence of a homolog of the electrically conductive pilin of *G. sulfurreducens* (e-pilin GSU1496) in *G. ferrihydriticus* genome [12,38] allows us to predict the production of highly conductive pili by *G. ferrihydriticus*. At the same time, genome analysis of ‘*Ca.* C. alkaliaceticus’ revealed the determinants of poorly conductive, ‘long’ type IV pili in this organism (see above). The detection of multiple cellular appendages connecting cells of *G. ferrihydriticus*, ‘*Ca.* C. alkaliaceticus’ and magnetite particles altogether support the assumption that the DIET mechanism is involved in syntrophic interactions between these organisms, for which magnetite could serve as an insoluble extracellular electron carrier when the cell ratio increases in favor of ‘*Ca.* C. alkaliaceticus’. The e-pili of *G. sulfurreducens* could be the main determinant of this DIET mechanism, while the ‘non-conductive’ pili-like structures of ‘*Ca.* C. alkaliaceticus’ could serve to attach the cells to each other and to spatially arrange conductive magnetite particles, which in turn could provide for additional electronic connections between the syntrophic partners.

Previous physiological studies of DIET were performed mostly with *Geobacter metallireducens* as the model electron-donating partner for DIET because this bacterium plays an important role in methanogenic environments such as anaerobic digesters [39,40] and terrestrial wetlands [41] as the syntrophic partner for methanogens. Studies of DIET have suggested that *c*-type cytochromes and electrically conductive pili (e-pili) facilitate electron transport from *G. metallireducens* to the electron-accepting partner [42,43,44]. It was suggested that outer surface *c*-type cytochromes and e-pili are involved in electron uptake by *G. sulfurreducens* in DIET-based co-cultures with *G. metallireducens* [42,44,45]. On the other hand, DIET can also be realized between different species through abiotic conductive materials, such as magnetite [46,47], granular activated carbon, biochar, carbon cloth, and some other materials [48,49]. Our experiment with the syntrophic association of ‘*Ca*. C. alkaliaceticus’ and *G. ferrihydriticus* growing on the surface of magnetite allow us to suggest that bacteria probably use both cell-to-cell and cell-to-magnetite strategies for the DIET. We were lucky to obtain the possibility of visualizing the biofilm formation on the co-culture. The growth of the syntrophic association on the magnetite surface gave clear advantages in cell yield to the obligate syntroph, which is atypical for this type of association. At the same time, all attempts to cultivate ‘*Ca*. C. alkaliaceticus’ separately from electron-accepting partners in the presence of magnetite failed. This may be caused by the need to alter the initial redox potential of magnetite to allow its interaction with the EET-driving proteins of ‘*Ca*. C. alkaliaceticus’. In such a case, the Fe-cycling partner organism, *G. ferrihydriticus*, could initiate the redox modification of magnetite and thus provide the EET to it from ‘*Ca*. C. alkaliaceticus’ cells. Furthermore, *G. ferrihydriticus*, which possesses a wide repertoire of EET-related complexes, could effectively scavenge electrons from both the magnetite particles and the ‘*Ca*. C. alkaliaceticus’ cells. The presumably higher efficiency of electron acceptance by *G. ferrihydriticus* cells compared to electron donation by ‘*Ca*. C. alkaliaceticus’ cells allow for the proliferation of a co-culture in which the syntrophic oxidizer outnumbers the partner organism (Figure 2b–d). Such a mode of DIET-driven syntrophy contrasts with the classical mechanism of IIET, which is likely to occur in the co-cultures of ‘*Ca*. C. alkaliaceticus’ with SRB, where the ratio of ‘*Ca*. C. alkaliaceticus’ cells to SRB never exceeded 1:10 [7].

## 4. Conclusions

Whole genome-resolved phylogenetic analysis placed ‘*Ca.* C. alkaliaceticus’ in the same cluster with acetogenic bacteria possessing Rnf complexes and using Na^+^-gradient for energy generation [11]. ‘*Ca.* C. alkaliaceticus’ possesses the Wood–Ljungdahl pathway for acetate oxidation and a bimodular respiratory system, including a Rnf complex that generates the sodium-motive force and a Na^+^-dependent F_1_F_0_-ATP synthase converting the transmembrane potential to ATP for energy conservation. In addition, Na^+^/H^+^ translocating pyrophosphatase, NADH:quinone oxidoreductase, and single- and multisubunit Na^+^/H^+^ antiporters can take part in the generation of the membrane potential. ‘*Ca.* C. alkaliaceticus’ can establish syntrophic associations whether by IIET mechanism using H_2_ and/or formate as the electron carriers or by DIET using conductive pili of its partner organism *G. ferrihydriticus* and electrically conductive minerals for EET. The possibility of the existence of the latter pathway stems from the formation of a complex biofilm where ‘*Ca.* C. alkaliaceticus’ and *G. ferrihydriticus* cells are interconnected with many extracellular appendages. The obtained data indicate a significant metabolic similarity between ‘*Ca.* C. alkaliaceticus’ and its phylogenetic relative ‘*Ca.* S. acetioxidans’ [5,8,9]. However, ‘*Ca.* C. alkaliaceticus’ revealed several remarkable features (DIET, genome mobility, and genetic immune systems) that expand our knowledge of the newly proposed class *Dethiobacteria*, the deep lineage in the phylum Firmicutes_D.

## Data Availability

The whole genome sequence, as well as sample and sequencing project information, were deposited in Genbank under accession numbers CP054699, SAMN15165809, and PRJNA638041 for genome, Biosample, and Bioproject, respectively.

## Supplementary Materials

The following supporting information can be downloaded at: https://www.mdpi.com/article/10.3390/life13102084/s1, Figure S1: Sequence alignment of CRISPR-associated proteins Cmr5 and the subunit Cmr5 (HUE98_14335) of ‘*Ca.* C. alkaliaceticus’; Table S1: Genome and environmental features of ‘*Ca.* C. alkaliaceticus’; Table S2: Structure of CRISPR-Cas locus of ‘*Ca.* C. alkaliaceticus’; Supplementary File S1: Genomic islands, including 108.9 kb of bacteriophage-related regions and 89 IS-element-related ORFs.
