# Supplementary material for: Genomic Insights into Syntrophic Lifestyle of ‘Candidatus Contubernalis alkaliaceticus’ Based on the Reversed Wood–Ljungdahl Pathway and Mechanism of Direct Electron Transfer
Source: Life (Basel). 2023 Oct 20;13(10):2084. doi: 10.3390/life13102084 (PMC10608574; doi:10.3390/life13102084)
Supplement: Supplementary file 1 [file life-13-02084-s001.zip › Supplementary Material.pdf]

Supplementary Material

Genomic insights into the carbon and energy metabolism of an obligately syntrophic alkaliphilic bacterium '*Candidatus* Contubernalis alkalaceticum'

Evgenii N. Frolov[1], Sergey N. Gavrilov[1], Stepan V. Toshchakov[2], Daria G. Zavarzina[1]

[1]Winogradsky Institute of Microbiology, Federal Research Center of Biotechnology, Russian Academy of Sciences, 60 let Oktjabrja pr-t, 7, bld. 2, 117312 Moscow, Russia

[2]National Research Centre "Kurchatov Institute", Akademika Kurchatova Sq., 1, 123182 Moscow, Russia

These authors contributed equally to this work.

Corresponding author: evgenii_frolov_89@mail.ru

Table S1. Genome and environmental features of '*Ca.* C. alkalaceticum'.

FEATURE	DESCRIPTION
**NCBI taxonomy**	Domain *Bacteria*<br>Phylum *Firmicutes*<br>Class *Clostridia*<br>Order *Clostridiales*<br>Family *Syntrophomonadaceae*<br>Genus *Candidatus Contubernalis*<br>Species *Candidatus Contubernalis alkalaceticum*<br>Type strain Z7904^T
**GTDB taxonomy***	Domain *Bacteria*<br>Phylum *Firmicutes_D*<br>Class *Dethiobacteria*<br>Order ND**<br>Family ND**<br>Genus ND**<br>Species ND**
**Biosample ref**	SAMN15165809 (NCBI)
**Collection date**	August 1997
**Geographical location name**	Russia, Tyva Republic, Khadyn soda lake
**Geographical coordinates**	51.358096 N, 94.517211 E
**Sequencing project ref**	PRJNA638041 (NCBI)
**Sequencing method**	Illumina MiSeq, Oxford Nanopore GridION
**Sequencing center**	NRC "Kurchatov Institute"
**Assembly method**	Unicycler v.0.4.8; CLC Genomics Workbench v.10.0
**Coverage**	89x (Illumina), 170x (Oxford Nanopore)
**Number of replicons**	1
**Finishing level**	Finished
**Genome size, bp**	3558951
**GC content, %**	41.2
**Genes**	3516
**Pseudogenes**	65
**RNA genes**	68
**rRNA**	13 (4 operons and one isolated 5S rRNA)
**tRNA**	51
**ncRNA**	4
**GI number**	19
**GI length (% share)**	414209 (11.6)
**GI gene number (% share)**	494 (14.05)
**Mobile elements (IS)**	89
**complete IS-related ORFs**	62
**partial ORFs**	5
**pseudogene ORFs**	9
**unknown**	48
**Number of different IS families**	36
**Prophage regions**	5
**intact**	2
**partial**	3
**total length (kb)**	108.9

Table S2. Structure of CRISPR-Cas locus of '*Ca.* C. alkalaceticum'.

LOCUS TAG	GENE	CDD database	SYSTEM TYPE (SUBTYPE)	STRAND	BEST BLASTP HIT (NR)	IDENTITY	E-VALUE	ACC
HUE98_14325	TIGR02710 family CRISPR-associated protein	cd09747	III	-	*Syntrophomonadaceae bacterium*	50%	1e-153	NLC07785.1
HUE98_14330	type III-B CRISPR module RAMP protein Cmr6	cd09661	III-B	-	*Desulfosporosinus* sp. Tol-M	43%	3e-108	KGP75449.1
HUE98_14335	type III-B CRISPR module RAMP protein Cmr5	cd09749	III-B	-	*Desulfosporosinus* sp. BRH_c37	50%	6e-29	KUO70795.1
HUE98_14350	type III-B CRISPR module RAMP protein Cmr4	COG1336	III-C	-	*Bacillus alveayuensis*	61%	3e-123	WP_044895091.1
HUE98_14355	type III-B CRISPR module-associated protein Cmr3	pfam09700	III-B	-	*Bacillus* sp. HMF5848	49%	2e-125	WP_125905557.1
HUE98_14360	type III-B CRISPR-associated protein Cas10/Cmr2	cd09679	III	-	*Bacillus* sp. HMF5848	44%	3e-159	WP_125905556
HUE98_14365	type III-B CRISPR module RAMP protein Cmr1	COG1367	III-B	-	*Bacillus* sp. HMF5848	44%	2e-77	WP_125905555.1
HUE98_14370	CRISPR-associated endonuclease Cas2	cd09725	I,II,III,V	-	*Pelotomaculum* sp. PtaB.Bin104	74%	5e-39	OPX90215.1
HUE98_14375	type I-B CRISPR-associated endonuclease Cas1	cd09722	I-B	-	*Thermincola*	68%	4e-167	WP_013121320.1
HUE98_14380	CRISPR-associated protein Cas4, pseudogene with internal stop	pfam01930	I	-	*Firmicutes bacterium*	59%	8e-68	HHU51258.1
HUE98_14385	CRISPR-associated helicase Cas3'	COG1203	I	-	*Firmicutes bacterium*	43%	0	MTI85228.1
HUE98_14390	CRISPR-associated protein Cas5	cd09692	I-B	-	*Firmicutes bacterium*	59%	7e-97	MTI85229.1
HUE98_14395	type I CRISPR-associated protein Cas7b	pfam05107	I-B/I-C	-	*Firmicutes bacterium*	65%	1e-154	MTI85230
HUE98_14400	hypothetical CRISPR-associated protein TM1802	cl21533	I-A/I-B	-	*Firmicutes bacterium*	43%	0	MTI85231.1
HUE98_14405	CRISPR-associated endoribonuclease Cas6	COG1583	I,III,IV	-	*Firmicutes bacterium*	58%	1e-100	HHY36210.1
HUE98_14410	CRISPR-associated protein Csm6	cd09742	III-A	-	*Desulfofundulus thermosubterraneus*	54%	1e-142	WP_072868410

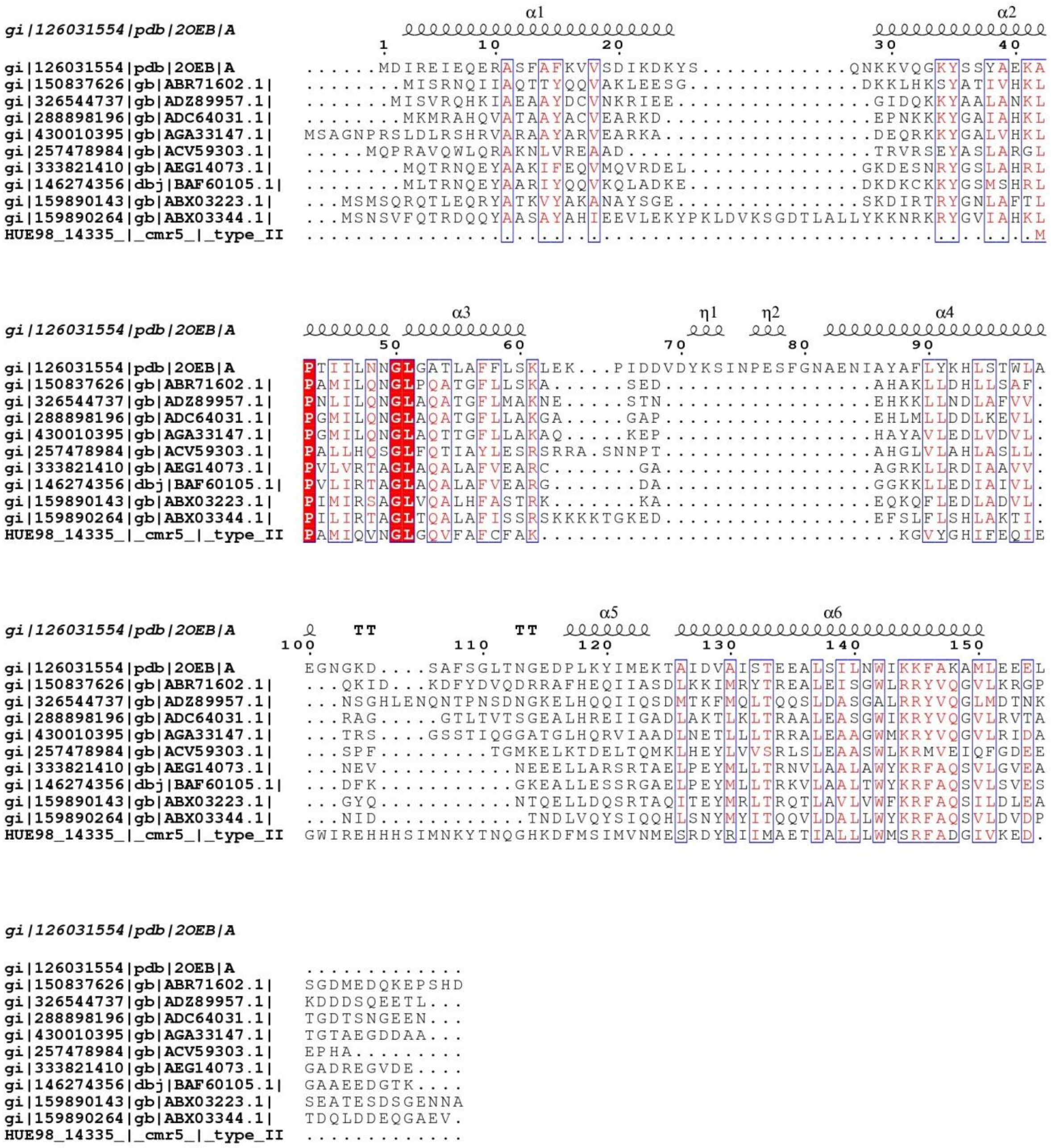

Figure S1. Sequence alignment of CRISPR -associated proteins Cmr5 and the subunit Cmr5 (HUE98_14335) of ‘*Ca.* C. alkalaceticum’.
